# Penis auto-amputation and chasm of the lower abdominal wall due to advanced penile carcinoma: a case report

**DOI:** 10.1186/1752-1947-5-574

**Published:** 2011-12-12

**Authors:** Dimitrios Baltogiannis, Konstantinos Zotos, Stavros Tsambalas, Dimitrios Giannakis, Nikolaos Sofikitis

**Affiliations:** 1 Department of Urology, Ioannina University School of Medicine, 45110 Panepistimioupolis, Ioannina, Epirus, Greece

## Abstract

**Introduction:**

Penile cancer is uncommon. When penile cancer is left untreated, at an advanced stage it can have tragic consequences for the patient.

**Case presentation:**

Our case report does not concern a new manifestation of penile cancer, but an interesting presentation with clinical significance that emphasizes the need to diagnose and treat penile cancer early. It is an unusual case of a neglected penile cancer in a 57-year-old Greek man that led to auto-amputation of the penis and a large chasm in the lower abdominal wall. The clinical staging was T4N3M0 and our patient was treated with a bilateral cutaneous ureterostomy, chemotherapy and radiotherapy. Our patient died 18 months after his first admission in our clinic.

**Conclusions:**

Emphasis must be placed on early diagnosis and treatment of penile cancer, so further development of the disease can be prevented.

## Introduction

Penile cancer accounts for less than 1% of all cancers in men [1]. It is a relatively rare squamous cell carcinoma (SCC), which usually originates in the epithelium of the inner prepuce and glans. Invasive carcinoma of the penis begins as an ulcerative or papillary lesion, which may gradually grow to involve the entire glans or shaft of the penis [2]. Primary dissemination is via lymphatic channels to the femoral and iliac nodes. Distant metastases are clinically apparent in less than 10% of cases, and may involve the lungs, liver, brain or bones. With regard to the diagnosis and staging, for penile cancer assessment of the primary lesion, regional lymph node disease and the possibility of distant metastases are all required [3]. The assessment is based on physical examination results, a lesion biopsy, ultrasound and ultrasound-guided fine-needle aspiration biopsy (FNAB) for non-palpable nodes, an abdominal computed tomography (CT) scan, a chest X-ray and additionally a bone scan in symptomatic patients who are classed as M1.

Treatment depends on the staging and varies from laser surgery (Tis and Ta) to partial or total penis amputation (T2, T3, T4) with or without inguinal lymphadenectomy (nodal metastases or not) and radiotherapy/chemotherapy if needed [3-6]. Although it is considered to be one of the few solid tumors that have a high curative rate, patients tend to delay seeking medical attention; this is mostly due to embarrassment, fear of emasculation, ignorance and personal neglect. A search through the literature revealed only seven other cases, none as severe as the one presented below [7,8].

## Case presentation

**Figure 1:**
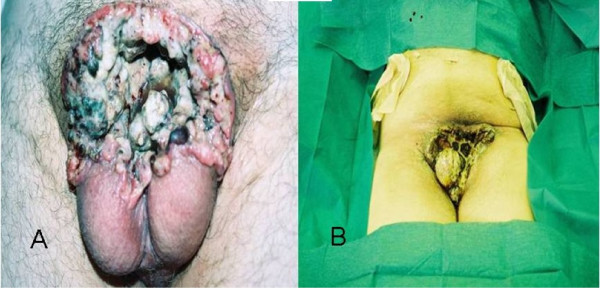
**Chasm of lower abdominal wall after penile auto-amputation before surgical treatment (A), and after surgery treatment (B)**. (Α) The penis is completely absent and there is a chasm in the lower abdominal wall that makes it possible to see parts of the lower pelvis and the spermatic cords, the destroyed base of the corpora cavernosa and the residual stump of the urethra. The scrotum and the testicles were stiff and possibly invaded by the cancer. In the chasm margins, we could detect hemorrhagic and necrotic areas. (Β) A bilateral cutaneous ureterostomy with Gibson incision was performed in order to protect the corroded tissues from further urine impregnation.

A 57-year-old Greek man was referred to our facility with pain, hemorrhage and a gangrenous smell due to a so-called wound on his penis. A physical examination revealed the complete absence of his penis and a large chasm in the lower abdominal wall, which made it possible to see parts of the lower pelvis, such as the spermatic cords, the destroyed basis of the corpora cavernosa and the residual stump of the urethra. The scrotum and the testicles were stiff and were possibly invaded by the cancer. In the chasm margins, we could detect hemorrhagic and necrotic areas (Figure 1A). The inguinal lymph nodes were palpable, hard and mobile. Our patient was in a good general condition and his body temperature was normal. From his medical history, he had discovered a lesion in his inner prepuce 18 months before. He had requested medical advice at a private health center concerning that lesion. According to his recollection, a biopsy had been taken and he was diagnosed as having penile cancer (this biopsy could not be found, as he did not ask for a copy of it at the time and the private health center failed to track our patient's data as he was never hospitalized there). The physicians at the time suggested he should undergo a partial penectomy, but he refused and stopped seeking medical treatment.

**Figure 2:**
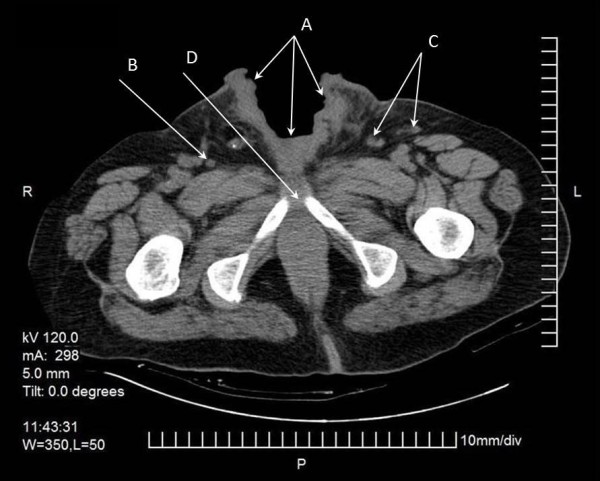
**Abdominal image from computed tomography (CT) scan**. The chasm of the lower abdominal wall (arrow A) and the inguinal lymph nodes bilateral can be identified (arrows B and C). The abdominal CT scan also showed lymph nodes of pathological numbers and sizes bilateral in the iliac vessels and inguinal areas, as well as an erosion of the pubic bone (arrow D).

The lesion slowly progressed, eventually involving the whole penis. He could not specify the exact time his penis sloughed off completely. He was not circumcised. Standard laboratory test results showed that his values were within normal limits except for a small rise in white blood cell count (14,750 cells/μL) and microcellular anemia (hemoglobin = 9.8 g/dL, hematocrit = 31.2%). A chest X-ray did not show any remarkable findings. An abdominal computed tomography (CT) scan showed lymph nodes of a pathological size and number, bilateral in the iliac vessels and inguinal areas as well as an erosion of the pubic bone (Figure 2). We proceeded with a chest CT scan, which did not show any distant metastases or lymph nodes. On the first day of his hospitalization, we obtained biopsies from the chasm margins and identified a poorly differentiated SCC. The clinical staging was T4N3M0 and our patient was treated with chemotherapy and regional radiotherapy. We also performed a bilateral cutaneous ureterostomy, with a Gibson incision in order to protect the corroded tissues from further urine impregnation (Figure 1B). From a combination of regional radiotherapy and bilateral cutaneous ureterostomy, total dryness of the wound was achieved. During his extended hospitalization, he presented with deep vein thrombosis in the right shin vein and seizures that were attributed to small ischemic brain strokes after a brain CT scan. Debulking and flap coverage of the wound was not considered possible, firstly because of deep vein thrombosis, epileptic seizures and his poor general condition increased the risk from operation and secondly the size of the chasm combined with very poor vascularization of the region (a topical angiography was performed). Gradually, our patient developed depression, denial of feeding and loss of weight. He died 18 months after his first admission and six months after his last follow-up admission to our clinic.

## Discussion

Carcinoma of the penis in developed countries accounts for about 0.4% of all malignancies in men. Penile carcinoma occurs most commonly in the sixth decade of life [9].

The risk factors are phimosis, chronic inflammatory conditions (for example, balanoposthitis) and treatment with sporalene and ultraviolet photochemotherapy [10]. The most common issue at presentation is the lesion itself. It may appear as an area of induration or erythema, ulceration, a small nodule, or an exophytic growth. Phimosis may obscure the lesion and result in a delay in seeking medical attention. In fact, 15% to 50% of patients delay seeking medical attention for at least a year [11]. Delays in diagnosis and initiation of therapy can affect survival and may lead to tragic and unusual cases of total penis auto-destruction [3,8].

Careful palpation of the inguinal area is mandatory because more than 50% of patients show enlarged inguinal nodes. Anemia and leukocytosis may be present in patients with a long-standing disease or an extensive local infection. A biopsy of the primary lesion is mandatory to establish the diagnosis of malignancy. Treatment varies depending on the pathology as well as the location of the primary lesion and the positive or negative nodal metastases [3,4,12,13].

Patients who have an inoperable disease and bulky inguinal metastases are often treated with chemotherapy. The four chemotherapeutic agents that have demonstrated activity against penile carcinoma are: bleomycin, methotrexate, cisplatin and 5-fluorouracil [14,15].

The percentage of five-year survival rates for patients with node-negative disease ranges from 65% to 90%. For patients with positive inguinal nodes this rate decreases to 30% to 50%, and for patients with positive iliac nodes it decreases to less than 20%. In the presence of soft-tissue or bony metastases, no five-year survivors have been reported. Squamous cell carcinoma accounts for 98% of all penile cancers. Sporadic cases of melanoma, basal cell carcinoma, and Paget's disease have all been reported. These lesions tend to be radiosensitive [3-5,7].

## Conclusions

We consider this case to be an interesting one with clinical significance, emphasizing the need to diagnose and treat penile cancer early so that further development of the disease is prevented.

## Consent

The person described in the case report has died. Written informed consent for publication from the patient's next-of-kin could not be obtained despite all reasonable attempts. The case is important to public health and every effort has been made to protect the identity of our patient. There is no reason to believe that our patient's next-of-kin would object to publication.

## Competing interests

The authors declare that they have no competing interests.

## Authors' contributions

All authors contributed to the study and the preparation of the manuscript. All authors read and approved the final draft.
